# Evaluation of Rapid Blood Sample Collection in the Detection of Circulating Filarial Antigens for Epidemiological Survey by rWbSXP-1 Capture Assay

**DOI:** 10.1371/journal.pone.0102260

**Published:** 2014-07-15

**Authors:** Lawrence Ansel Vishal, Y. Nazeer, Rajendran Ravishankaran, Natarajan Mahalakshmi, Perumal Kaliraj

**Affiliations:** Centre for Biotechnology, Anna University, Guindy, Chennai, Tamil Nadu, India; Centro de Pesquisa Rene Rachou/Fundação Oswaldo Cruz (Fiocruz-Minas), Brazil

## Abstract

**Background:**

Lymphatic filariasis is a neglected tropical disease leading to profound disfiguring causing socio economic burden in the tropics. Current diagnosis strategies available during field surveys and epidemics are based on traditional microscopic detections and a few antigen/antibody assays. We have compared different sampling methodologies and standardized the highly sensitive and reliable r*Wb*SXP-1 antigen detection assay to our new sampling methodology.

**Methodology:**

Samples collected as serum, whole blood, whole blood on filter paper and whole blood on microscopic slides from patients belonging to various clinical groups of filariasis [endemic normal(EN), chronic pathology(CP), microfilaraemic(MF) and non-endemic normal(NEN)] were collected and standardized the r*Wb*SXP-1 antigen detection assay using monoclonal antibody raised against r*Wb*SXP-1 protein. The whole blood collected on microscopic slide based sampling method was employed in the field and the presence of circulating filarial antigen (CFA) was assessed using the r*Wb*SXP-1 assay.

**Principal Findings:**

The sampling methods were compared and no significant difference was observed for the detection of CFA (MF, P = 0.304, EN, P = 0.675, CP, P = 0.5698, NEN, P = 0.4494). Further the optimized sampling method was utilized to collect the 1106 samples from Polur, Tiruvannamalai. The r*Wb*SXP-1 assay gave 98 antigen positive results whereas the microscopic method gave only 17.

**Conclusions:**

Four sampling methodologies were analyzed and the new sampling methodology of whole blood collected on microscopic slide was found to be convenient for the detection of CFA using r*Wb*SXP-1 antigen detection assay. The 1106 samples from Polur were collected using the new method. The r*Wb*SXP-1 antigen assay perceived a 7.32% increased result which was read as false negatives on the conventional microscopic staining method. This new sampling methodology coupled with the r*Wb*SXP-1 antigen assay can be used in epidemiological surveys for lymphatic filariasis and the same sampling methodology can be expanded to other antigen based high affinity assays.

## Introduction

Human Lymphatic filariasis (LF) is a neglected tropical infectious parasitic disease caused by lymph dwelling nematodes *Wuchereria bancrofti*, *Brugia malayi*, & *Brugia timori*. Amongst which *Wuchereria bancrofti is* the most dominant driving force for the infection in the sub continent [1]. The parasite is transmitted by arthropod vectors belonging to the genera *Culex*, *Anopheles*, *Aedes*, and *Manosonia*. *Culex quinquefasciatus* is accountable for 50% of transmission throughout the world [2], [3]. The infection is acquired at early childhood but realization of the diseased state is often only after a late onset of disfiguring morbidity. The Global Program to Eliminate Lymphatic Filariasis (GPELF) initiated by The World Health Organization targets to eliminate the disease by the year 2020, but to accomplish such a feat, sensitive and reliable diagnostic tests are required for early clinical detections, field evaluations and post therapy monitoring [4]. The diagnosis of the infection during surveys and post infection is done by the thick microscopic smear stained with JSB or giemsa stains [5], which is always subjected to chance when working with infected individuals with low microfilaria (mf) density. Such cases require agitation of the parasite by using anti-filarial drugs, thus leading to underestimation of mf prevalence rates in epidemiological surveys [3], [6], [7]. Whereas the circulating filarial antigen (CFA) detections in Og4C3, ICT card, and other similar antigen, antibody tests prove to be more sensitive, efficient, quick, easy and cost-effective [8]. The ICT filarial antigen test and the Og4C3 assay are based on detection of adult worm antigens [9], [10]. Later, detection assays utilizing the mf stage antigens were developed using various targets such as r*Wb*Shp-1, *Bm*14 and r*Wb*SXP-1 [11], [12], [13].

In this study we have used a similar CFA detection by using the r*Wb*SXP-1 antigen sandwich ELISA. The *Wb*-SXP-1 protein is expressed in the mf stage of the parasite. The r*Wb*SXP-1 antigen used in our assay was seen to be highly sensitive and specific as a diagnostic tool and will provide an early detection for the filarial infection [14], [15]. The study samples were collected through four different methods and detected by using the r*Wb*SXP-1 antigen detection assay with monoclonal antibodies raised in mouse. The monoclonal antibody 1F6H3 which is highly reactive with brugian and bancroftian parasites were used as capture antibody and polyclonals raised in rabbit for r*Wb*SXP-1 was used for detection as described previously [12]. We have evaluated the samples for the detection of CFA with the assay and derived at an optimized detection parameter for the r*Wb*SXP-1 antigen detection assay to the samples collected as whole blood on microscopic slides, thereafter we have surveyed the filarial endemic village Polur of Tiruvannamalai district in Tamil Nadu, India using this new and easier mode of sampling and the results were compared with the conventional microscopic staining detection.

## Materials and Methods

### Ethical statement

All protocols were followed in accordance with the guidelines of the Committee for the Purpose of Control and Supervision of Experiments on Animals (CPCSEA), Animal Welfare Division, Ministry of Environment and Forests, Government of India. The animal experiments have been approved by the Institutional Animal Ethics Committee, Centre for Biotechnology, Anna University, Chennai, Tamil Nadu, India “(Ref No-CBT/AU/IAEC/01/2013)”. Mice were housed in 12-h night–day cycle at controlled temperature of 24°C and food ad libitum. For the collection of blood samples from human individuals appropriate permissions were attained from the Department of Public Health and Preventive Medicine, Govt. of Tamil Nadu “(Ref No-26433/VCII/SI/2012)” before collecting the samples and conducting the field survey. The procedures followed were in accordance with the guidelines issued by the same department. Written and oral consent was taken from individuals before conducting the sampling. The institutional review board at the Centre for Biotechnology, Anna University, India also approved the protocols.

### Human clinical samples for standardization

Samples from asymptomatic microfilaremics (MF) (n = 20) were collected from endemic regions of Tamil Nadu, India. The patients were identified as per the traditional thick smear microscopic data provided by the Department of Public Health and Preventive Medicine and Zonal Entomology Team, Vellore. Endemic normal (EN) (n = 20) samples were collected from individuals of the endemic villages with no mf present in thick smear microscopic test. Chronic pathology (CP) (n = 20) subjects with visible clinical symptoms of lymphedema were collected from Vellore and Tiruvannamalai districts. All the samples were collected by four different sampling methods. Samples were collected as 7 ml venous blood and 5 ml from it was used for separating the serum. The remaining 2 ml blood was stored separately for the whole blood analysis and 30–50 µl blood was used for the assay [16]. A single finger prick was made using a sterile steel lancet (Ghia Surgiblades Pvt. Ltd., Mumbai, India) and 20 µl of blood was absorbed on Whattman filter paper no. 3 in triplicates, air dried and stored. Samples were prepared as described by Hoti et. al. for the filter paper sample [17], [18]. A new single 2–3 mm deep finger prick was made using the common diabetic test lancing device and sterile needles (*Glucopro, NIPRO Corporation, Japan*). A smear (100–150 µl) was made on a glass slide, dried and stored in slide boxes. After 3–4 hours the smear was resuspended with 150 µl of sterile phosphate buffered saline (PBS). The sample was transferred into sterile 1.5 ml eppendorf tubes and stored. This sample was used for the whole blood collected on microscopic slide samples and used for r*Wb*SXP-1 assay experiments, 30 µl of this sample was used for the assay. All the samples were stored in −80°C until further use. The samples were collected in between 21:00 hrs and 23:00 hrs. Non-endemic normal (NEN) (n = 10) samples from healthy volunteers residing in non endemic area was provided by Dr. Murray Selkirk, Professor of Biochemical Parasitology, Division of Cell and Molecular Biology, Imperial College London, London.

### Sample Survey

The study was conducted in Polur village of Tiruvannamalai district, Tamil Nadu, India under the supervision of corresponding healthcare officials from the Department of Public Health and Preventive Medicine, Govt. of Tamil Nadu. The survey samples were collected by using the lancing device and sterile needles (*Glucopro, NIPRO Corporation, Japan*). The samples were collected on glass slides in duplicates. One slide was used for the traditional thick smear microscopic detection by staining with JSB stain [5]. The other slide was used for resuspending the smear with 150 µl PBS and stored as described previously.

### Recombinant Protein Expression and Purification

The recombinant clone of *Wb*SXP-1 (Gen Bank Acc. No: AF098861) cloned in pRSETb was used in the study. The construct was transformed into salt-inducible *Escherichia coli* GJ1158 cells [19] and the recombinant antigen was expressed as a fusion protein with polyhistidine tag. The culture was grown up to ∼0.6 optical density (OD) and the expression was induced by NaCl to a final concentration of 250 mM. The culture was allowed to grow for another 4 h at 36°C post induction. The cells were further pelleted by centrifugation and solubilised in binding buffer (100 mM Tris, 100 mM NaH2PO4, 200 mM NaCl, pH8.0). Cells were disrupted by sonication, centrifuged and the supernatant was subjected for purification. HIS-tagged r*Wb*SXP-1 was purified by using Immobilized Metal Affinity Chromatography (IMAC)(Amersham, GE) on chelating sepharose matrix under non-denaturing conditions [20]. The expression and purification was analyzed on 12% sodium dodecyl sulphate polyacrylamide gel electrophoresis (SDS-PAGE) and confirmed by immunoblotting with anti-HIS antibody [21].

### Immunoreactivity of r*Wb*SXP-1 protein

Immunoreactivity of the r*Wb*SXP-1 was checked with clinical sera of MF, EN, CP and NEN using pooled sera from 10 human serum samples. The purified r*Wb*SXP-1 antigen was separated on 12%SDS-PAGE, electrotransferred onto nitrocellulose membrane. After blocking overnight with 5% skimmed milk the membrane strips were incubated individually with (1∶50) pooled sera from the different clinical groups. The bound antibodies were probed with alkaline phosphatase labelled goat antihuman IgG, and colour was developed with NBT/BCIP chromogenic substrate complex (ThermoFisher Scientific, Rockford, IL) [22].

### Generation of antibodies for ELISA assay

Monoclonal antibody 1F6H3 synthesized earlier in our laboratory were used for the assay and 1F6H3 was identified to have more reactivity to native and recombinant mf antigen. It also had higher affinity and avidity to recombinant antigen [12]. The polyclonals were prepared by immunizing laboratory-bred rabbits with purified r*Wb*SXP-1 protein [23]. Purified recombinant protein (250 µg) was emulsified in Freund's complete adjuvant and administered subcutaneously; consequently 125 µg of antigen was given in Freund's incomplete adjuvant. 10–13 days post final immunization the animals were bled and tested for immunoreactivity against the r*Wb*SXP-1 by ELISA. The reactivity of the purified polyclonal was confirmed by western blots [12].

### Detection of circulating filarial antigen by r*Wb*SXP-1 sandwich ELISA

Purified 1F6H3 monoclonal antibodies (1 µg/well in PBS) were coated separately on to each well of the high-binding 96 well microtiter ELISA plates (Thermo Scientific, Waltham, MA) and incubated at 4°C overnight as capture antibody. Blocking of the wells is done by 200 µl of 5% skimmed milk and incubating the plates at 37°c for 1 hr. Simultaneous PBS and PBST (0.05% Tween20 dissolved in PBS) washes were done followed by addition of corresponding patient sample (sera, whole blood, filter paper and slide method) and incubation for 2 hours at room temperature. Detection of CFA is attained by adding rabbit anti-r*Wb*SXP-1 polyclonal antibody followed by goat anti-rabbit HRP conjugated secondary antibodies (Santacruz biotechnology, USA) with 1 hour incubation after adding each antibody. The plates were washed extensively and the bound peroxidase activity was detected using tetramethyl benzidine substrate. The reaction was stopped by using 1 M H_2_SO_4_ solution. The results were articulated as mean absorbance at 450 nm for each group ± standard deviation (OD±SD). Samples were identified as positive when the optical density was higher than the mean OD+3SD value of 10 NEN control sera [11].

### Statistical Analysis

Statistical analysis for all the experiments was done using the GrapPad Prism software version 5.0 (GraphPad Software, San Diego, CA). Comparison of mean values was done using non-parametric Mann-Whitney analysis for two mean values, and one way analysis of variance (ANOVA) was used for comparison of more than two values. P≤0.05 was considered to be statistically significant.

## Results

### r*Wb*SXP-1 Protein expression and purification

**Figure 1:**
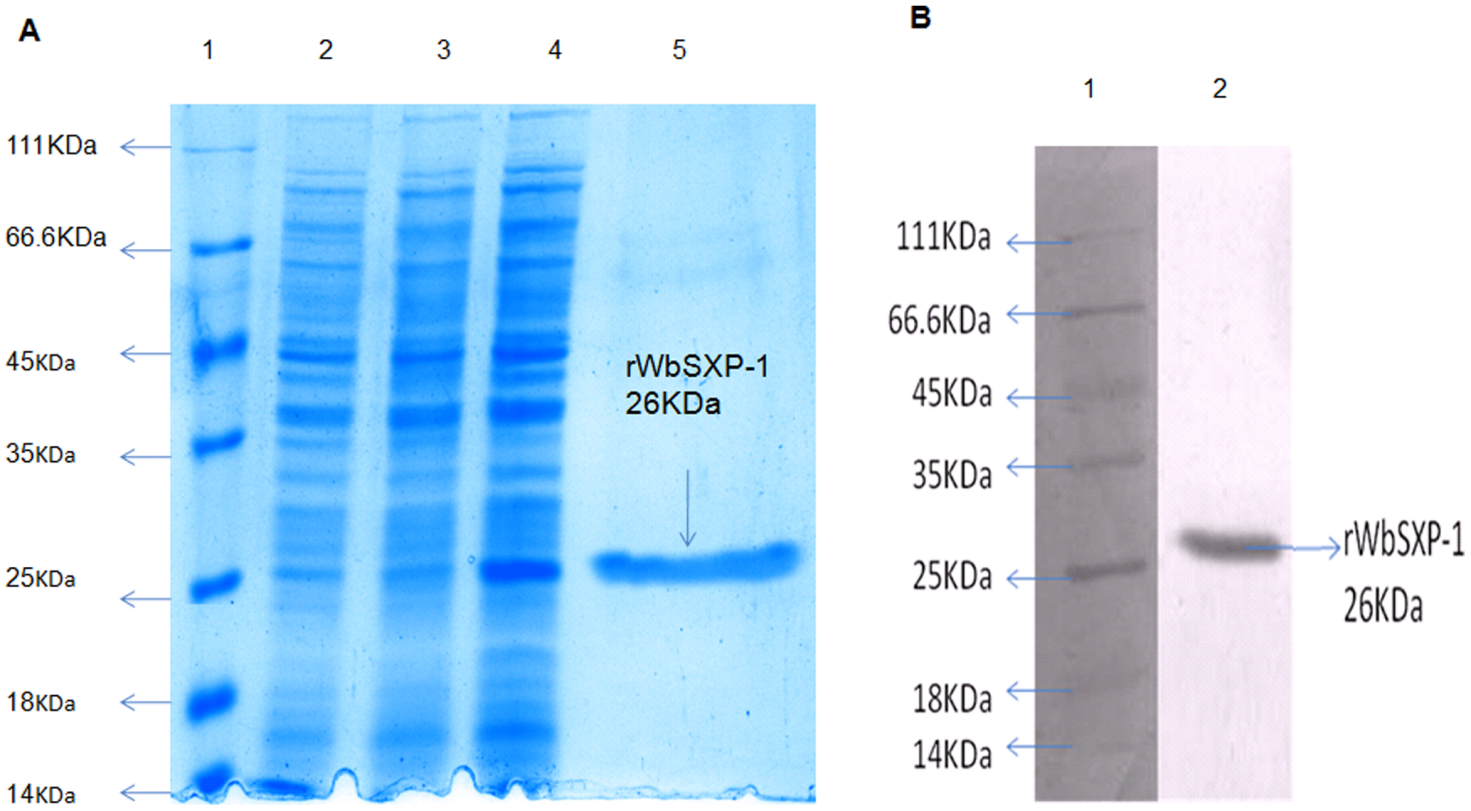
Expression and purification of r*Wb*SXP1 protein. (**A**). Expression of r*Wb*SXP-1 was performed by inducing salt inducible *E.coli* GJ1158 with 250 mM NaCl. The cell lysate was electrophoresed on a 12.5% polyacrylamide gel under reducing conditions and stained with Coomassie brilliant blue. Lane 1: molecular weight markers; Lane 2:vector induced; Lane 3: r*Wb*SXP-1 uninduced; Lane 4:r*Wb*SXP-1 induced; Lane 5: r*Wb*SXP-1 purified protein. (**B**). Immunoreactivity of purified r*Wb*SXP-1 with Anti-histidine antibody(dilution 1∶10,000). Lane 1 : molecular weight marker; Lane 2 : Purified r*Wb*SXP-1.

The recombinant *Wb*-SXP-1 protein was over expressed in salt inducible *E.coli* GJ1158, using NaCl at a final concentration of 250 mM. The protein was obtained at a molecular weight of 26 KDa (Figure 1A) and confirmed by western blotting with anti histidine antibody after purification by IMAC (Figure 1B).

### Immunoreactivity of r*Wb*-SXP-1 antigen

**Figure 2:**
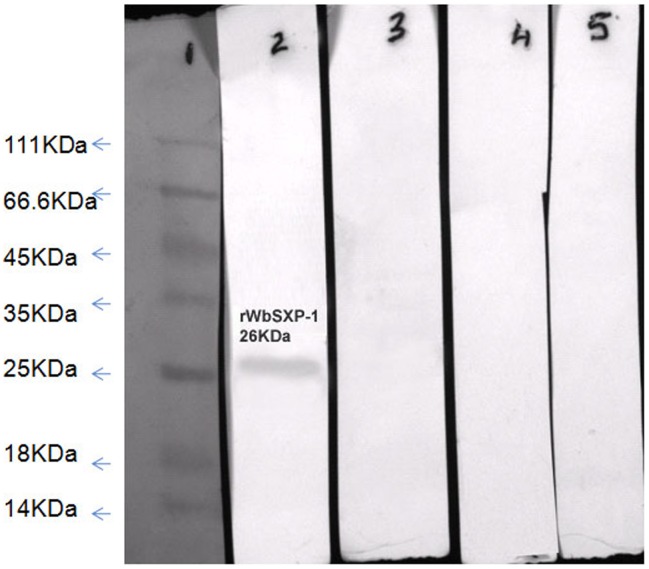
Human immune response to r*Wb*SXP-1. Immunoreactivity of r*Wb*SXP-1 with different clinical sera of lymphatic filariasis. 2 µg (approx) of purified r*Wb*SXP-1 was electrophoresed on 12% SDS-PAGE and transferred onto nitrocellulose membrane, blots were probed with pooled sera from MF, EN, CP and NEN individuals. High reactivity with pooled MF sera was seen with No reactivity with EN, CP and control NEN sera. Lane 1 : molecular weight marker; Lane 2 : MF; Lane 3: EN; Lane 4: CP; Lane 5: NEN.

The immunoreactivity of r*Wb*-SXP-1 was analysed by checking the reactivity of the purified antigen against pooled sera samples of MF, EN, CP and NEN (samples n = 10). The antigen showed reactivity with MF sera and no reactivity with the EN, CP and NEN sera (Figure 2).

### Sampling method Optimization

Four types of samples were collected from each individual and the samples were used for the standardization of the assay. The MF, EN, CP and NEN samples were identified by the traditional microscopic method and visible clinical symptoms. Samples such as serum separated from the venous blood, whole blood samples, samples collected on filter paper and samples from the slide method were collected as described earlier. Separate assays were performed for the EN, MF, CP and NEN groups with the above samples and the results were taken for optimising the r*Wb*SXP-1 antigen detection assay based on the slide method.

**Figure 3:**
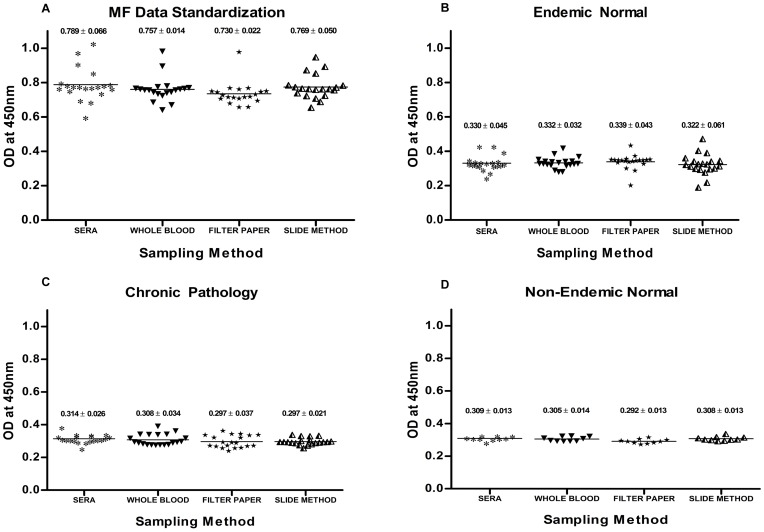
Sampling method standardization with r*Wb*SXP-1 assay. Samples from (**A**) MF, (**B**) EN, (**C**) CP, (**D**) NEN were analysed. Samples collected as sera, whole blood, on filter paper and by the new slide method are shown. Each data point represents individual absorbance from the four different types of samples. The horizontal bars represents the mean value of each group. The values are expressed as mean ±SD.

The MF (Figure 3A), CP (Figure 3B), EN (Figure 3C) and NEN (Figure 3D) samples when analyzed with the r*Wb*SXP-1 antigen detection assay showed no significant difference in their OD values, when comparing the slide method samples with the sera and whole blood samples (Table 1). Four EN samples were detected as positive by our assay. These four samples were positive for all the four types of sampling methods. The positivity of these four samples was confirmed by Signal-MF filarial detection kit (Span Diagnostics, Gujarat, India) [24].

**Table 1:** Analysis of different sampling method with r*Wb*SXP-1 assay.

	MF (Mean ± SD)	EN (Mean ± SD)	CP (Mean ± SD)	NEN (Mean ± SD)
Sera	0.789±0.066	0.330±0.045	0.314±0.026	0.309±0.013
Whole Blood	0.757±0.014	0.332±0.033	0.308±0.034	0.305±0.014
Filter Paper	0.730±0.022	0.339±0.043	0.297±0.037	0.292±0.013
Slide Method	0.7689±0.050	0.322±0.061	0.297±0.021	0.308±0.013
Sera vs Slide significance	P = 0.3040(ns)	P = 0.6750(ns)	P = 0.5698(ns)	P = 0.4494 (ns)

**Figure 4:**
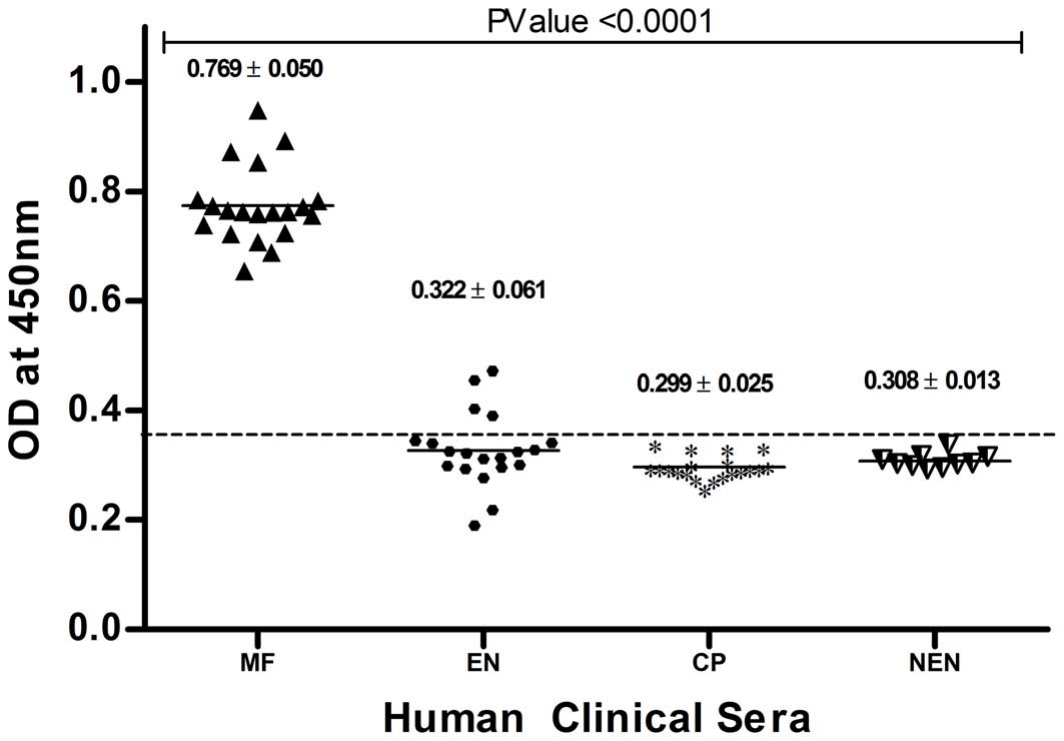
Standardized r*Wb*SXP-1 assay for the slide method of samples. Four different clinical groups MF (n = 20), EN (n = 20), CP (n = 20) and NEN (n = 10) were used for the assay. The MF samples had high significance with a P value of <0.0001. The horizontal bars represents the mean value of each group. The values are expressed as mean ±SD and shown in the graph. The cut off value of 0.3477 is calculated by NEN+3SD. Samples with OD values above the cut off are considered as r*Wb*SXP-1 positive.

Using this data a standard plot for r*Wb*SXP-1 detection assay for the slide method samples was developed (Figure 4). The antigen levels in MF group samples were significantly higher when compared with EN, CP and NEN groups with a p value of <0.0001(Table 2). The cut off value of 0.3477 for the antigen positive samples were calculated and assigned using the formula NEN+3SD. Since the samples collected on microscopic slide were comparable with the samples collected by other methods, the same was used for field evaluation of antigen detection assay.

**Table 2:** Standardized r*Wb*SXP-1 assay with slide method samples.

Clinical Group(By Traditional method)	No. of Samples	CFA Positive (Percentage detection)	MEAN	SD
Microfilaraemic (MF)	20	20 (100%)	0.768	±0.050
Endemic Normal (EN)	20	4 (20%)	0.322	±0.061
Chronic Pathology (CP)	20	0 (0%)	0.297	±0.021
Non Endemic Normal (NEN)	10	0 (0%)	0.308	±0.013

### Field Survey

The endemic village Polur from Tiruvannamalai district of Tamil Nadu was taken for the field survey. Samples were collected in duplicates by using the optimized slide method of sample collection. A total of 1106 samples were collected of which 525 (47.5%) were females. The age of the individuals selected for sample collection was between 3–85 years. Further detection by traditional microscopic staining method and r*Wb*SXP-1 antigen detection from samples collected by slide method was performed (Table 3).

**Table 3:** Survey details for Polur Village.

Mean age (years; range)	Total No.	Female (%)	No. of positives (Microscopic Method)	No of CFA positive (r*Wb*SXP-1 assay)
5 (03–10)	98	40.8	0	0
17 (11–20)	295	40.3	0	4
26 (21–30)	252	46.8	2	22
38 (31–40)	160	55	7	32
44 (41–50)	197	48.2	4	21
53 (51–60)	60	0.6	3	12
69 (61–85)	44	65.9	1	7
TOTAL	1106	47.5	17 (1.54%)	98 (8.86%)

### r*Wb*SXP-1 Detection Assay for evaluation of field samples

**Figure 5:**
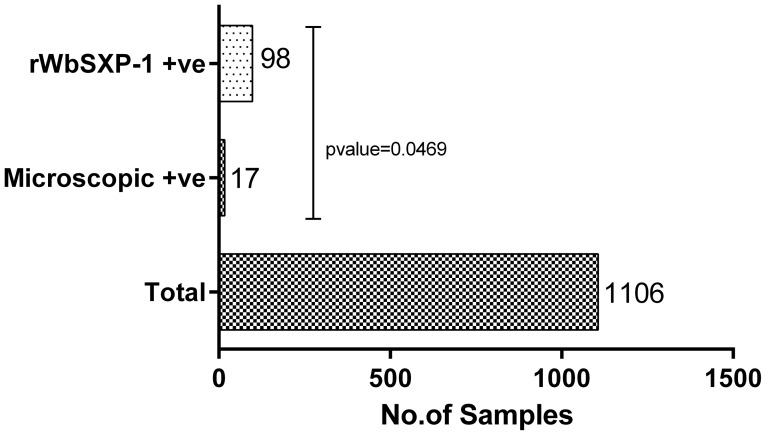
r*Wb*SXP-1 assay for Polur samples collected by new slide method. The samples collected in Polur, Tiruvannamalai, Tamil Nadu were assayed by the r*Wb*SXP-1 assay and by thick smear microscopic detection after staining with JSB stain. A total of 98 antigen positive samples were detected by r*Wb*SXP-1 assay, whereas the microscopic detection gave only 17 positives. The 17 positive samples detected by the microscopic method were positive in the assay also. The Antigen detection was significant with the microscopic detection with a P value of 0.0469.

r*Wb*SXP-1 detection assay for the samples collected by slide method was carried out and the samples with a OD value higher than the cut off (0.3477) was considered as positive. A total of 17/1106 (1.54%) samples were identified as mf positive individuals by the conventional microscopic staining method, the mf density was within the range of 40–380 mf/ml with a geometric mean of 5.73 in the infected blood. Whereas 98/1106 (8.86%) was identified as positive by the r*Wb*SXP-1 assay. The 17 positive samples identified by traditional method was also CFA positive for the r*Wb*SXP-1 assay amongst the additional 81 (7.32%) samples (Figure 5, Table 3). No mf was detected in any of the night blood smears examined from subjects who were negative in the r*Wb*SXP-1 assay.

## Discussion

The effective detection and control of filariasis is anchored on the identification of infection and assessment of the disease. This was thought to be achieved completely by the traditional microscopic staining method, although light was shed into the fact that microscopic detection is not completely reliable and feasible as there were difficulties in sampling, in terms of, time of collection, segregating the large volume of samples, and most importantly the finding of mf in blood smear was based on chance. It depends on factors such as the stage of infection, collection time and density of mf in the patient. Later detections based on CFA were introduced and the Immunochromatographic card test (ICT) and Og4C3 ELISA became the limelight for filarial detection [9], [25], while this CFA detection had their advantages, sensitivity and storage issues were noted. Other detection assays based on filarial antibody detections (*Wb*SXP, *Bm*14, *Bm*R1) were also developed [15], [24], [26]. Although antibody detections were developed for the detection of nematode infections, the antigen based detections proved to be more informative than antibody methods [27], due to the fact that endemic controls often harbour high titres of antibody than the infected people [28]. Amongst these CFA detection assays a very high affinity *Wb*SXP-1 assay was introduced by us using monoclonal and polyclonal antibodies specific to the parasite [12].

In all the above techniques the prime issue faced is the inability to collect and process large volumes of samples from the field during surveys or an epidemic. Most sampling is done by means of finger pricking for the microscopic staining and from collecting venous blood to separate the serum for detection of CFA. Commercially available detection methods use field samples collected in the form of filter paper or the whole blood for detection of CFA [16]. The collection of samples for detection of CFA from the serum has its restriction as the collection, segregation and storage of such samples are very hard. A recently developed rapid test (Alere Filariasis Test Strip) for detecting CFA in human blood detects the infection within 10 minutes, whereas gives late positives after 24 hours [16]. Primarily during large scale field surveys and endemic outbreaks when the sample volume will be very high and in thousands, it is time consuming to wait for 10 minutes at each home and conduct tests as per the manufacturer's instruction. Thus when working with large volumes of samples, collection of samples from the field and evaluation at a later time is required.

Therefore in this study we have made an attempt to develop an easy, quick, reliable, cost effective and sensitive sampling and detection technique. Using this method we can conduct large scale surveys and detection of the infection in quick notice, and with minimal hardships. The slide method described in our study is a simple technique that requires not more than 3 people for conducting the sampling and detection. It is a less invasive, time saving, inexpensive technique that does not require a trained specialist for the collection and storing of the samples [4], [6]. Also since the needle and the lancing devices are put into use, it is less likely that the pricking process will leave painful injuries on the fingers of the individuals tested. Unlike night blood surveys, the samples can be collected and stored with ease throughout the day, since it is CFA detection.

For the current study we have used the r*Wb*SXP-1 assay , previously described by us which has high sensitivity with clinical sera of *Wb* (90.8%) and *Bm* (91.4%), and has no cross reactivity with *Oncocerca volvulus* infected sera [12], [24]. The purified r*Wb*SXP-1 antigen showed reactivity with MF pooled sera and no reactivity with CP, EN and NEN sera while checked on immunoblots. The four different sample collection modes when analyzed with the r*Wb*SXP-1 assay have shown similar or mild variations in their optical density, which makes it clear that the samples collected by the slide method has sensitivity on a par with sera samples. While comparing the P value of sera based r*Wb*SXP-1 detection versus samples collected by the slide method it was seen that for the MF (P = 0.3040), EN (P = 0.6750), CP (P = 0.5698), NEN (P = 0.4494) samples the values were not significant. It was also noted that the OD values of the samples analyzed are not significant with the OD values of whole blood detections (Table 1). The filter paper methodology of sampling has shown less sensitivity for the r*Wb*SXP-1 assay, similar incompetency of the filter paper samples with respect to sensitivity and specificity were previously reported [18], [29]. During our optimization we have found that four individuals who have been characterised as endemic normal by traditional microscopic method were having the r*Wb*SXP-1 antigen in their system. This clearly entails that the traditional microscopic method failed to prove its reliability by providing with false negative results. A low density of mf in the system or prepatent infection can lead to CFA in the system but lack mf in the blood smear or any clinical symptoms [24], similar findings were reported with microscopic detections earlier [18], [30], [31]. This cryptic infection which is overlooked during field surveys [32], [33] and control of infection leads to the failure of MDA and eradication of filariasis. Such cases that have escaped the detection will further proliferate the infection by substituting as fresh reservoirs for transmission. Thus a conclusion can be drafted that the slide method can be used as an alternative for the surveying and infection analysis using r*Wb*SXP-1 assay.

In the second part of our study we have conducted a field survey of Polur village, from Tiruvannamalai, Tamil Nadu with the new slide based sampling method and analyzed the same with r*Wb*SXP-1 assay. A total of 1106 samples were collected among which 581 males and 525 females were analyzed by both, traditional microscopic staining method and the r*Wb*SXP-1 assay. In our results we have found that 1.54% samples were only identified as mf positive in the conventional thick smear microscopic staining method whereas 8.86% was identified as positive by the r*Wb*SXP-1 assay. The 17(1.54%) samples identified as mf positive were also CFA positive in the r*Wb*SXP-1 assay. The 7.32% increased positivity in our assay could be due to cryptic or occult infection in the patients. Since CP patients can eliminate the SXP antigen from the system owing to the Th1 response they possess, they were detected as negatives in our assay [12], [34], [35].

## Limitations

To confirm the statement of cryptic situation, qPCR measures could give an insight, whereas to do the same genomic dna extracted from mf is required, which was not present in the slides or the blood obtained from antigen positive individuals. Similarly in regions where co-infection of loasis is seen, this method might not be suggested as cross reactivity with *Loa loa* serum of SXP antibody detection was noted earlier. However since loasis is not co-endemic with brugian and bancroftian infections in most endemic countries, this could not cause any problem [24].

## Conclusions

To summarize four sampling methods were analyzed and an easy, cost effective method which is less invasive, mass survey friendly and reliable was identified and optimised for the high affinity r*Wb*SXP-1 antigen detection assay. Further the endemic village of Polur, Tiruvannamalai, Tamil Nadu was surveyed with the new sample collection method and assayed using the optimized r*Wb*SXP-1 assay. This methodology of sample collection and assay can be further employed in large scale surveys and detections owing to the merits it poses towards sampling and storage, without compromising the sensitivity and reliability of the detection. The survey can be further performed in other endemic areas for filarial infections and also can be used to identify the cryptic infections amongst the population. It can also be used as a yardstick to assess the state and volume of infection in EN populations where the infection is claimed to be absent. Thereafter MDA programmes can be administered long before clinical manifestations or a widespread endemic occurs. The sampling methodology can be further diversified into other parasitic infections but this cannot be discussed in detail until further research is done on this subject.

## Supporting Information

Dataset S1 **ELISA standardization data.** The ELISA data used for comparing different methods of sampling and standardizing the rWbSXP-1 assay to the slide based sample collection method.
(XLSX)

Dataset S2 **Polur rWbSXP-1 assay data.** Field evaluation of the rWbSXP-1 assay using samples collected from Polur, Tiruvannamalai using the slide based sampling method.
(XLSX)
